# The association between diabetes knowledge and medication adherence among patients in Saudi Arabia

**DOI:** 10.12669/pjms.42.2.12752

**Published:** 2026-02

**Authors:** Khalid Alayed, Nouf Alsubaie, Manal Altwaim, Mohammed Almutairi, Norah Alawlah

**Affiliations:** 1 Khalid Alayed Assistant Professor, Department of Medicine, College of Medicine, King Saud University Riyadh, Saudi Arabia; 2 Nouf Alsubaie College of Medicine, King Saud University Riyadh, Saudi Arabia; 3 Manal Altwaim College of Medicine, King Saud University Riyadh, Saudi Arabia; 4 Mohammed Almutairi College of Medicine, King Saud University Riyadh, Saudi Arabia; 5 Norah Alawlah College of Medicine, King Saud University Riyadh, Saudi Arabia

**Keywords:** Diabetes, Diabetes knowledge, Glycemic control, Medication adherence

## Abstract

**Objectives:**

To assess the relationship between knowledge regarding diabetes and medication adherence among diabetic patients.

**Methodology:**

A cross-sectional study was conducted at the diabetes, primary care and internal medicine clinics in King Khalid University Hospital, Riyadh, Saudi Arabia, over a period of six months (July 2024 - December 2024). Data were collected using an electronic questionnaire incorporating two validated scales: the simplified diabetes knowledge scale and Morisky Medication Adherence Scale. Analysis was performed using SPSS version 26.0, applying the Chi-square test for categorical variables and Spearman’s rank correlation to assess the relationship between knowledge and adherence. Statistical significance was set at p ≤ 0.05, with 95% confidence intervals.

**Results:**

The mean age was 51.1± 16.5 years, with 39.6% over 60. Males constituted 52.9% of the sample, and 66.9% were married. The majority (61.5%) had Type-II diabetes and good glycemic control (HbA1c < 8) (51.1%). A weak correlation was found between diabetes knowledge and medication adherence (r = 0.118, p = 0.115). Diabetes knowledge was good in 50.7% with a positive correlation with educational attainment (p = 0.000) and employment status (p = 0.042). Medication adherence was low in 32%, medium in 36.7% and high in 31.3%. Higher adherence rates were observed in patients with Type-II diabetes (p = 0.010) and those who have good glycemic control (p = 0.006).

**Conclusion:**

Although diabetes knowledge is important for promoting adherence, it is not sufficiently effective on its own to guarantee compliance. Targeted educational interventions, particularly for those with lower education, unemployment, or a recent diagnosis, may enhance adherence, improve outcomes and reduce the burden of diabetes in Saudi Arabia.

## INTRODUCTION

Diabetes is considered an epidemic worldwide as declared by the International Diabetes Federation (IDF). Approximately 537 million people worldwide had diabetes in 2021 and that number is expected to rise to 783 million by 2024. Furthermore, the IDF estimates that 17.7% of Saudi Arabia’s population has diabetes mellitus.^1^,^2^ According to the World Health Organization (WHO), Saudi Arabia had the second-highest prevalence of DM in the Middle East and ranked seventh globally.^3^

Patient compliance with diabetes pharmacological agents (insulin, oral hypoglycemic medications and injectable agents)^4^,^5^ plays a crucial role in achieving successful diabetes management.^6^ According to the Centers for Disease Control and Prevention (CDC), medication adherence among diabetic patients is associated with improved clinical outcomes, reduced mortality and morbidity rates, lower hospitalization rates and decreased healthcare costs at a national level^7^. However, the literature shows a substantial gap in the correlation between patients’ adherence to medication and their knowledge of the disease.^8^,^9^

In light of the critical role that medication adherence plays in the treatment of diabetes mellitus, this study aimed to evaluate the correlation between diabetes knowledge and medication adherence among diabetic patients in Riyadh, Saudi Arabia. Additionally, we aimed to explore how demographic factors influence these variables. By assessing the educational needs of patients with diabetes, healthcare workers can customize their communication and support strategies more effectively.

## METHODOLOGY

A cross-sectional study was conducted at King Khalid University Hospital (KKUH), Riyadh, Saudi Arabia, over a period of six months (July 2024 - December 2024). The sample size of this study was determined using a single proportion formula, based on reported prevalence of T2DM in Saudi Arabia which is estimated at 17.7%^1^,^2^,^7^ according to previous literature. With 95% confidence interval and a 5% margin of error, the minimum required sample size was calculated to be 224 participants. To account for an anticipated non-response rate of 35%, an additional 78 participants were added, resulting in a targeted sample size of 302 diabetic patients attending the diabetes clinics, primary care clinics and internal medicine clinics. A convenience sampling technique was employed, whereby eligible patients who attended these clinics during the study period and agreed to participate were included in the sample. An electronic questionnaire was used as a tool for data collection using two validated scales.

The Simplified Diabetes Knowledge Scale (SDKS) and Morisky Medication Adherence Scale (MMAS-8). Reliability of the SDKS and MMAS-8 in previous research has shown Cronbach’s alpha values of ≥ 0.7 and above, indicating good internal consistency.^10^-^12^ Investigators collected data through an online survey via QR code which was distributed among visitors in the waiting area of diabetes clinics, primary care clinics and internal medicine clinics.

### Ethical approval and Consent:

The study was approved by the Institutional Review Board of King Saud University (E-23-8407; dated: July 6, 2025). Written informed consent was obtained from patients before participation.

### Research instrument:

The validated Arabic version of SDKS was used to assess participants’ knowledge. The scale consists of 20 items, of which 18 items are general questions and two items are specific to insulin-treated diabetics. The items address diet, exercise, glycated hemoglobin (HbA1c), foot care, regular follow-up and information about diabetes complications. Responses follow a “True/False/Don’t Know” format. Participants who answered more than 65% of the questions correctly (i.e. 13/20 or 12/18 correct answers) were considered to have good knowledge of diabetes mellitus.^10^ The validated Arabic version of MMAS-8 was used to assess participants’ adherence to medications. The scale consisted of eight items; seven items were answered with a yes or no and one item with a 5-point Likert scale. The scores of the MMAS-8 range from 0 to 8. A score below six indicates low adherence, a score between 6 and < 8 indicates medium adherence and a score of 8 indicates high adherence.^11^-^13^

### Statistical analysis:

Data analysis was conducted using SPSS software version 26.0 (IBM Corp, Armonk, NY, USA). Descriptive statistics, including frequency, percentages, means and standard deviations, were employed to express categorical and quantitative variables. The Chi-square test was utilized for the comparison of categorical variables. Furthermore, the Spearman rank correlation test was applied to assess the relationship between knowledge and medication adherence. Statistical significance was determined using a p-value threshold of ≤ 0.05.

## RESULTS

Out of 307 participants, 29 (9.4%) were excluded due to incomplete or missing data, giving rise to a final sample of 278 participants. The mean age of the participants was 51.1 ± 16.5 years, with 52.9% males and 66.9% married. In terms of diabetes type, Type-II diabetes was more prevalent (61.5%). The duration of diabetes varied among participants; 38.1% had been diagnosed for more than 20 years and 15.8% had been living with diabetes for less than five years. The remaining demographic characteristics are listed in (Table-I).

**Table-I:** Demographic Characteristics of participants (n=278).

Item	Categories	No.	%
Age range (y) Mean ± SD (51.1± 16.5) Range (18-85)	18-39	77	27.7%
	40-49	42	15.1%
	50-59	49	17.6%
	>60	110	39.6%
Gender	Male	147	52.9%
	Female	131	47.1%
Marital status	Single	49	17.6%
	Married	186	66.9%
	Divorced/ Widow	43	15.5%
Nationality	Saudi	270	97.1%
	Non-Saudi	8	2.9%
Education	No schooling	25	9%
	Primary school & Middle	45	16.2%
	Matriculation	59	21.2%
	College graduate	149	53.6%
Employment	Employed	87	31.3%
	Retired	76	27.2%
	Unemployed	115	41.5
Type DM	Type-I	107	38.5%
	Type-II	171	61.5%
Duration of disease (y)	< 5	44	15.8%
	5 - 9	39	14%
	10 - 14	54	19.4%
	15 - 19	35	12.6%
	> 20	106	38.1%
BMI	Normal	81	29.1%
	Overweigh	90	32.4%
	obese	107	38.5%
The last measurement Hb1AC was more than 6 months ago	Yes	74	26.6%
	No	204	73.4%
Hb1AC1	Uncontrolled ≥8	135	48.6%
	Controlled <8	143	51.4%
SDKS	Poor knowledge	137	49.3%
	good knowledge	141	50.7%
MMAS	Low	89	32%
	Medium	102	36.7%
	High	87	31.3%

Knowledge regarding diabetes, as assessed by SDKS, revealed that 49.3% of participants exhibited poor knowledge, while 50.7% demonstrated good knowledge. Medication adherence, evaluated using the Morisky Medication Adherence Scale (MMAS), indicated that 32% of participants had low adherence, 36.7% had medium adherence and 31.3% had high adherence (Table-I).

### Medication Adherence and Knowledge Assessment:

Based on MMAS findings, 35.3% of respondents reported occasionally forgetting to take their antidiabetic medications, while 25.9% indicated that there were instances within the preceding two weeks when they did not adhere to their prescribed regimen. Furthermore, 19.1% acknowledged having reduced or discontinued their medication without prior consultation with a healthcare provider, attributing this decision to a perceived deterioration in their condition. The responses to other adherence items are demonstrated in (Table-II). Concerning knowledge assessment using SDKS, a majority of participants (77.3%) correctly responded to the first item (SDKS1), suggesting a solid understanding of diabetes. However, significant knowledge gaps were identified in other areas; for instance, in response to item three (where the correct answer is “False”) (SDKS3), 57.2% of participants provided an incorrect response (Table-III, Table-IV).

**Table-II:** Description of diabetic’s Adherence by the Morisky 8-Item Medication Adherence Scale.

Items in MMAS Cronbach’s Alpha (0.681)	Yes N (%)	No N (%)
1. Do you sometimes forget to take your antidiabetic pills?	98 (35.3%)	180 (64.7%)
2. Over the past 2 weeks, were there any days when you did not take your antidiabetic medicine?	72 (25.9%)	206 (74.1%)
3. Have you ever cut back or stopped taking your medication without telling your doctor because you felt worse when you took it?	53 (19.1%)	225 (80.9%)
4. When you travel or leave home, do you sometimes forget to bring along your educations?	70 (25.2%)	208 (74.8%)
5. Did you take your antidiabetic medicine yesterday? *	260 (93.5%)	18 (6.5%)
6. When you feel like your blood glucose is under control, do you sometimes stop taking your medicine?	38 (13.7%)	240 (86.3%)
7. Do you ever feel hassled about sticking to your antidiabetic treatment plan?	65 (23.4%)	213 (76.6%)
8. How often do you have difficulty remembering to take all your antidiabetic medication?		
a. Never	161 (57.9%)	
b. Almost never	46 (16.5%)	
c. Sometimes	59 (21.2%)	
d. Quite often	6 (2.2%)	
e. Always	6 (2.2%)	

The MMAS-8© Scale, content, name and trademarks are protected by US copyright and trademark laws. Permission for use of the scale and its coding is required. A license agreement is available from MMAR, LLC., www.moriskyscale.com.

**Table-III:** description of diabetic’s knowledge level by simplified diabetes knowledge scale.

Item SDKS	I don’t Know N (%)	False N (%)	True N (%)
SDKS1*	25 (9%)	38 (13.7%)	215 (77.3%)
SDKS2	73 (26.3%)	136 (48.9%)	69 (24.8%)
SDKS3	73(26.3%)	171 (61.5%)	34 (12.2%)
SDKS4	64 (23%)	196 (70.5%)	18 (6.5%)
SDKS5	88 (31.7%)	119 (42.8%)	71 (25.5%)
SDKS6*	27 (9.7%)	78 (28.1%)	173 (62.2%)
SDKS7	50 (18%)	171 (61.5%)	57 (20.5%)
SDKS8*	60 (21.6%)	36 (12.9%)	182 (65.5%)
SDKS9*	25 (9%)	16 (5.8%)	237 (85.3%)
SDKS10	13 (4.7%)	243 (87.4%)	22 (7.9%)
SDKS11*	125 (45%)	18 (6.5%)	135 (48.6%)
SDKS12	46 (16.5%)	50 (18%)	182 (65.5%)
SDKS13*	24 (8.6%)	16 (5.8%)	238 (85.6%)
SDKS14*	68 (24.5%)	11 (4%)	199 (71.5%)
SDKS15	128 (46%)	92 (33.1%)	58 (20.9%)
SDKS16*	83 (29.9%)	58 (20.9%)	137 (49.2%)
SDKS17 (n =221)	45 (16.2%)	159 (72.3%)	17 (7.5%)
SDKS18* (n =221)	26 (11.8%)	11 (5%)	184 (83.2%)
SDKS19*	7 (2.5%)	2 (0.7%)	269 (96.8%)
SDKS20	7 (2.5%)	8 (2.9%)	263 (94.6%)

“True” response indicated by *, otherwise the correct response is “False”

**Table-IV:** Frequency distribution of correct answers to knowledge questions.

Questions about diabetic knowledge N = 278	N (%)
The diabetes diet is a healthy diet for most people.	215 (77.3%)
Glycosylated hemoglobin (HbA1c) is a test that measures your average blood glucose level in the past week	136 (48.9%)
A kilo of chicken has more carbohydrates in it than a kilo of potatoes	171 (61.5%)
Orange juice has more fat in it than low-fat milk.	196 (70.5%)
Urine testing and blood testing are both equally as good for testing the level of blood glucose.	119 (42.8%)
Unsweetened fruit juice raises blood glucose levels.	173 (62.2%)
A can of a soft drink can be used for treating low blood glucose levels.	171 (61.5%)
Using olive oil in cooking can help prevent raised cholesterol in the blood.	182 (65.5%)
Exercising regularly can help reduce high blood pressure.	237 (85.3%)
For a person in good control, exercising does not affect blood sugar levels.	243 (87.4%)
Infection is likely to cause an increase in blood sugar levels	135 (48.6%)
Wearing shoes a size bigger than usual helps prevent foot ulcers.	50 (18%)
Eating foods lower in fat decreases your risk for heart disease.	238 (85.6%)
Numbness and tingling may be symptoms of nerve disease.	199 (71.6%)
Lung problems are usually associated with having diabetes.	92 (33.1%)
When you are sick with the flu you should test for glucose more often.	137 (49.3%)
Having regular check-ups with your doctor can help spot the early signs of diabetes complications.	269 (96.8%)
Attending your diabetes appointments stops you from getting diabetes complications.	8 (2.9%)
Among 154 patients using insulin N= 221	
High blood glucose levels may be caused by too much insulin.	160 (72.4%)
If you take your morning insulin but skip breakfast your blood glucose level will usually decrease.	184 (83.3%)

### Association Between Knowledge, Adherence and Demographic Characteristics:

The findings revealed a statistically significant correlation between educational attainment and knowledge levels (p = 0.000), indicating that individuals with higher educational qualifications tend to possess greater knowledge regarding diabetes management. Moreover, unemployed participants exhibited lower knowledge levels, with only 48.2% demonstrating adequate understanding, compared to 25.5% of employed participants (p = 0.042). Patients diagnosed with Type-II diabetes reported higher adherence rates (73.6%) compared to those with Type-I diabetes (39.3%, p = 0.010).

Furthermore, the duration of diabetes presented a trend toward significance concerning knowledge levels (p = 0.050), suggesting that individuals with a shorter duration of diabetes tend to have less knowledge than those with a longer duration. Additionally, patients with controlled diabetes had higher adherence rates (65.5%) compared to those with uncontrolled disease (34.5%, p = 0.006, Table-V). This study revealed a weak correlation between diabetes knowledge and medication adherence (r = 0.118).

**Table-V:** Association of knowledge and adherence with Demographic Characteristics.

Variables	SDKS			MMAS			
	Poor, N (%)	Good, N (%)	P value	Low, N (%)	Medium, N (%)	High, N (%)	P value
Age range (y)			0.066				0.227
18-39	28 (20.4%)	49 (34.8%)		24 (27.0%)	30 (29.4%)	23 (26.5%)	
40-49	22 (16.1%0	20 (14.2%)		19 (21.3%)	16 (15.7%)	7 (8.%)	
50-59	27 (19.7)	22 (15.6%)		17 (19.1%)	15 (14.7%)	17 (19.5%)	
>60	60 (43.8%)	50 (35.5%)		29 (32.6%)	41 (40.2%)	40 (46%)	
Gender			0.286				0.322
Male	68 (49.6%)	79 (56.0%)		51 (57.3%)	48 947.1%)	48 (55.2%)	
Female	69 (50.4%)	62 (44.0%)		38 (42.7%)	54 (52.9%)	39 (44.8%)	
Marital status			0.337				0.709
Single	20 (14.6%)	29 (20.6%)		18 (20.2%)	18 (17.6%)	13 914.9%)	
Married	93 (67.9%)	93 (66.0%)		59 (66.3%)	65 (63.7%)	62 (71.3%)	
Divorced/ Widow	24 (17.5%)	19 (13.5%)		12 (13.5%)	19 (18.6%)	12 (13.8%)	
Education			0.000*				0.445
No schooling	19 (13.9%)	6 (4.3%)		7 (7.9%)	6 (5.9%)	12 (13.8%)	
Primary & Middle	31 (22.6%)	14 (9.9%)		12 (13.5%)	21 (20.6%)	12 (13.8%)	
Matriculation	31 (22.6%)	28 (19.9%)		21 (23.6%)	21 (20.6%)	17 (19.5%)	
College graduate	56 (40.9%)	93 (66%)		49 (55.1%)	54 (52.9%)	46 (52.9%)	
Employment			0.042*				0.874
Employed	35 (25.5%)	52 (36.9%)		29 (32.6%)	30 (29.4%)	28 (32.2%)	
Unemployed	66 (48.2%)	49 (34.8%)		31 (34.8%)	48 (47.1%)	36 (41.4%)	
Retired	36 (26.3%)	40 (28.4%)		29 (32.6%)	24 (23.5%)	23 (26.4%)	
Type DM			0.158				0.010*
Type-I	47 (34.3%)	60 (42.6%)		35 (39.3%)	49 (48.0%)	23 (26.4%)	
Type-II	90 (65.7%)	81 (57.4%)		54 (60.7%)	53 (52.0%)	64 (73.6%)	
Duration of disease (y)			0.050*				0.395
< 5	27 (19.7%)	17 (12.1%)		11 (12.4%)	19 (18.6%)	14 (16.1%)	
5 to 9	24 (17.5%)	15 (10.6%)		12 (13.5%)	19 (18.6%)	8 (9.2%)	
10 to 14	25 (18.2%)	29 (20.6%)		22 (24.7%)	13 (12.7%)	19 (21.8%)	
15 to 19	19 (13.9%)	16 (11.3%)		12 (13.5%)	12 (11.8%)	11 (12.6%)	
> 20	42 (30.7%)	64 (45.4%)		32 (36%)	39 (38.2%)	35 (40.2%)	
BMI			0.303				0.126
Normal	37 (27%)	44 (31.2%)		23 (25.8%)	35 (34.3%)	23 (26.4%)	
Overweigh	41 (29.9%)	49 (34.8%)		34 (38.2%)	23 (22.5%)	33 (37.9%)	
Obese	59 (43.1%)	48 (34%)		32 (36%)	44 (43.1%)	31 (35.6%)	
Hb1AC			0.724				0.006*
Uncontrolled ≥8	68 (49.6%)	67 (47.5%)		48 (53.9%)	57 (55.9%)	30 (34.5%)	
Controlled <8	69 (50.4%)	74 (52.5%)		41(46.1%)	45 (44.1%)	57 (65.5%)	

## DISCUSSION

The increasing standardized Disability-Adjusted Life Year (DALY) rate for diabetes in Saudi Arabia highlights the importance of evaluating diabetes knowledge and medication adherence among individuals with diabetes. In the current study, diabetes knowledge was assessed using the Simplified Diabetes Knowledge Scale (SDKS), revealing that nearly half (49.3%) of the study participants were found to have poor knowledge about diabetes. The prevalence of poor knowledge varies across the literature. For instance, a substantially high prevalence of poor knowledge was found in a study done in Taif, Saudi Arabia^14^, where 78.4% of the study population was found to have poor knowledge. Conversely, studies conducted in Kuwait^15^ and Iraq^16^ reveal a lower prevalence of poor knowledge, at 41.1% and 21.1%, respectively. Such variability could be explained by the different assessment tools used to evaluate the patient’s knowledge; in these studies, the Michigan Brief Diabetes Knowledge and Michigan Diabetes Knowledge Test were used, while in our study, SKDS was used.

Our study demonstrated a positive correlation between the educational attainment of diabetic patients and their knowledge about diabetes, which was supported by studies performed in the Gulf region,^14^,^15^ the United States^17^, and Portugal.^18^ This may be attributed to the fact that a large proportion of the study participants had high educational levels. Additionally, employed participants had relatively high knowledge in comparison to unemployed participants, with only 25% of employed participants exhibiting poor knowledge. Our finding aligns with a study from South Africa, which showed that being employed was positively associated with higher diabetes knowledge.^19^ The observed finding may be explained by the fact that employment is also associated with higher socioeconomic status and educational attainment, both of which are linked to increased awareness and understanding of health conditions such as diabetes.

Moreover, the duration of diabetes was found to be significantly associated with patients’ knowledge about diabetes, where patients with a longer duration of diabetes were found to be more knowledgeable about diabetes. This finding was supported by previously mentioned studies in the literature.^15^,^18^ A possible explanation is the fact that patients living with diabetes for a long period of time are more likely to engage in repeated interactions with healthcare providers.

Our study shows that 32% of the patients had low adherence, 36,7% had medium adherence. The reason why they had low to medium adherence levels was not only because of poor knowledge, as there are multiple factors leading to this adherence, like following an unhealthy diet and stopping taking their medication, thinking their condition had improved. A Saudi study using the same assessment tool MMAS-8, found that nearly half of the study population 54.8% have low adherence and 34.5% medium adherence. They attributed the reasons to non-adherence to diabetes medication to non-adherance to the regular follow-ups at diabetes clinics, following unhealthy dietary practices, and non-compliance to medication instructions.^20^ Another study in Riyadh found 23%, 37% low to medium adherence respectively. They found multiple factors potentially associated with low adherence, which are behavioral factors, social influences, and knowledge gaps about diabetes.^21^ A study conducted in Eastern Province, Saudi Arabia, showed that 21.5%, 47.7% of patients had low and medium adherence levels, respectively. Furthermore, female gender was a significant factor linked to low medication adherence.^22^

The present study also showed a significant association between medication adherence and the type of diabetes, where patients with Type-II diabetes demonstrated higher medication adherence rates (73.6%) compared to those with Type-I diabetes (39.3%). There are several potential reasons for such an observation. The primary contributing factor is the complexity of the treatment regimens. Managing Type-I diabetes often requires many daily insulin injections or continuous insulin infusion via pumps, in addition to frequent blood glucose monitoring; hence, these management aspects would compromise adherence. On the other hand, people often manage Type-II diabetes with oral medications, which are generally easier to adhere to.

Although diabetes knowledge is a major influencer of effective self-management in diabetic patients, our study revealed a weak correlation between knowledge and medication adherence, highlighting that other factors such as psychological, cultural, and economic factors may play a major role in medication adherence. For instance, denial of the diagnosis, fear of hypoglycemia, diabetes-related distress, and the perception that medication is not necessary once patient symptoms are controlled are all psychological factors that may lead to poor medication adherence. When it comes to the cultural influence on medication adherence, reliance on traditional remedies may negatively impact patient adherence. Furthermore, medication cost and lack of free access to health care could influence medication adherence even if the knowledge is adequate. Other Saudi and regional studies align with our findings, which report a modest positive relationship between diabetes knowledge and adherence. For instance, a cross-sectional study located in Khobar found a weak-to-moderate and positive correlation (ρ = 0.221, p < 0.01) between diabetes knowledge and adherence.^8^ Similarly, a study in the Eastern province noted that patients with better knowledge were more adherent (rho = 0.425, p < 0.001).^22^ Furthermore, a cross-sectional study in Madinah showed overall medium-to-high levels of medication adherence mostly influenced by age, comorbidities, education level, physician satisfaction, and health self-awareness.^23^

These insights should prompt policymakers and healthcare providers to adopt a holistic patient-centered approach by addressing patients’ fears, beliefs, and cultural contexts. In primary care practice, this means incorporating structured diabetes self-management education (DSME) with curricula tailored to local cultural norms. Implementing a DSME-accredited program such as the DiaServ initiative in Riyadh has shown significant improvement in self-confidence to manage diabetes, glycemic control, and adherence while reducing complications and care costs.^24^ Incentives for team-based chronic care models and educating multidisciplinary teams (pharmacists, dietitians, nurses, and community health workers) to offer DSME are two ways that policymakers and healthcare executives can encourage this.

Finally, in order to integrate education through the system, legislators and health administrators should implement structural changes. This could entail creating national guidelines for patient education, requiring insurance coverage for diabetes education and counseling, and assisting with public awareness campaigns. Care delivery could be enhanced by incorporating pharmacist-led counseling and diabetes educators into primary care, with reimbursement depending on education and adherence results.

### Limitations:

First, the causal relationships among knowledge, adherence and disease outcomes might have been limited by the cross-sectional design. Second, the use of self-reported tools, such as the MMAS and SDKS, may have led to reporting bias. Future longitudinal studies are recommended to investigate these correlations over time and determine causation. Future research should explore personalized educational interventions aimed at enhancing diabetes understanding and adherence, especially among high-risk populations, including those with lower educational levels or unemployment. Furthermore, examining the influence of cultural and psychological variables among Saudi diabetic individuals on adherence habits may provide a deeper comprehension of the barriers to optimal diabetes care.

## CONCLUSION

The current study found a weak correlation between knowledge and adherence to medications among patients with diabetes. The majority of participants had poor knowledge and moderate adherence. Lower knowledge levels were observed among participants with lower educational attainment and those who were unemployed. These findings highlight the importance of targeted diabetes education programs to improve knowledge and medication adherence, particularly among vulnerable populations, such as unemployed individuals, those with lower educational attainment and patients newly diagnosed with diabetes. Healthcare providers should implement a patient-centered approach that addresses both knowledge gaps and psychosocial barriers. Policymakers should consider the implementation of initiatives that promote adherence and reduce the burden of diabetes in Saudi Arabia.
